# The expression of the T-box selector gene *midline* in the leg imaginal disc is controlled by both transcriptional regulation and cell lineage

**DOI:** 10.1242/bio.013565

**Published:** 2015-11-18

**Authors:** Pia C. Svendsen, Jae-Ryeon Ryu, William J. Brook

**Affiliations:** Genes and Development Research Group, Alberta Children's Hospital Research Institute, Department of Biochemistry and Molecular Biology, Cumming School of Medicine, University of Calgary, 3330 Hospital Drive NW, Calgary T2N4N1, Alberta, Canada

**Keywords:** Bmp, *Drosophila melanogaster*, T-box genes, Wnt, Limb pattern formation

## Abstract

The *Drosophila* Tbx20 homologs *midline* and *H15* act as selector genes for ventral fate in *Drosophila* legs. *midline* and *H15* expression defines the ventral domain of the leg and the two genes are necessary and sufficient for the development of ventral fate. Ventral-specific expression of *midline* and *H15* is activated by Wingless (Wg) and repressed by Decapentaplegic (Dpp). Here we identify VLE, a 5 kb enhancer that drives ventral specific expression in the leg disc that is very similar to *midline* expression. Subdivision of VLE identifies two regions that mediate both activation and repression and third region that only mediates repression. Loss- and gain-of-function genetic mosaic analysis shows that the activating and repressing regions respond to Wg and Dpp signaling respectively. All three repression regions depend on the activity of *Mothers-against-decapentaplegic*, a *Drosophila* r-Smad that mediates Dpp signaling, and respond to ectopic expression of the Dpp target genes *optomoter-blind* and *Dorsocross 3*. However, only one repression region is responsive to loss of *schnurri*, a co-repressor required for direct repression by Dpp-signaling. Thus, Dpp signaling restricts *midline* expression through both direct repression and through the activation of downstream repressors. We also find that *midline* and *H15* expression are both subject to cross-repression and feedback inhibition. Finally, a lineage analysis indicates that ventral *midline*-expressing cells and dorsal *omb-*expressing cells do not mix during development. Together this data indicates that the ventral-specific expression of *midline* results from both transcriptional regulation and from a lack of cell-mixing between dorsal and ventral cells.

## Abstract (precis)

**Summary:** Leg expression of the selector gene *midline* in *Drosophila* is controlled both by transcriptional regulation and by the lack of mixing between dorsal and ventral cells in the distal leg.

## INTRODUCTION

The Wnt family protein Wingless (Wg) and the Tbx20 class T-box transcription factors *midline* (*mid*) and *H15* are the key regulators of the patterning of the ventral region of the fly leg. Wg is secreted by a wedge of ventral cells in the leg imaginal disc and in the absence of Wg all ventral structures are lost and are replaced with a duplication of dorsal structures (Baker, 1988; Held et al., 1994). Ectopic Wg expression induces ectopic ventral fate (Struhl and Basler, 1993). The specification of ventral fate by Wg depends on the expression of *mid* and *H15,* which act as selector genes for ventral fate in the fly leg. *mid* and *H15* act redundantly in the development of ventral structures and are sufficient to transform some dorsal structures into ventral (Svendsen et al., 2009). Thus *mid* and *H15* are key regulators of ventral fate and defining how their expression is restricted to ventral cells is essential for understanding leg development.

**Fig. 1.**
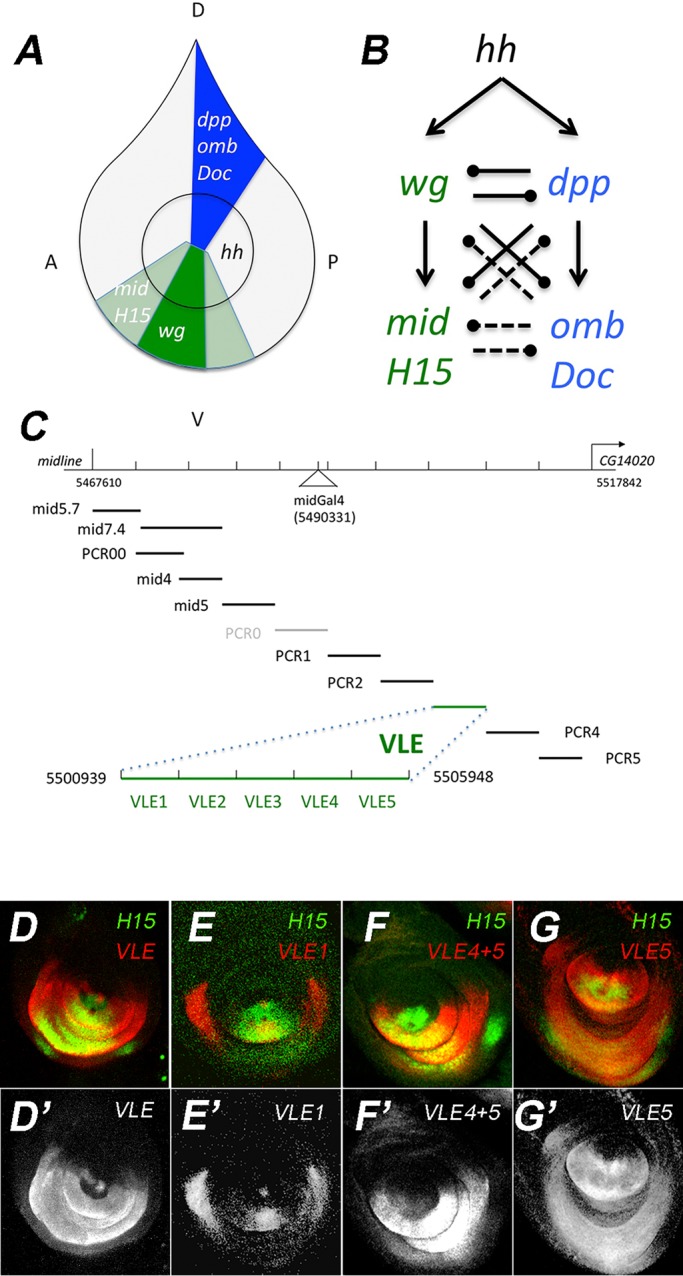
**Genetic pathway controlling D/V gene expression and identification of a *mid* leg enhancer.** (A) Diagram of the leg imaginal disc fate map. The dorsal (blue) and ventral (green) domains are labeled with genes of the Dpp and wg pathways required for development of those regions of the adult leg. (B) The genetic interactions that control D/V gene expression. Arrows indicate activation, clubs indicate repression, solid lines indicate the interaction has been demonstrated in both loss-of-function and gain-of-function assays, and dashed lines indicate that only gain-of-function has been demonstrated. (C) The genomic region 3′ to the *mid* coding region (CG6634, Flybase; Release 5.48 January 2013 *Drosophila* Genome Annotation). A previously identified enhancer-trap (NP2113, at genomic sequence coordinate 2L:5490331) is labelled *mid*Gal4. The fragments shown below the line were tested for leg enhancer activity in flies transformed with *lacZ* reporter constructs (with the exception of PCR0; see Discussion). Only VLE (ventral leg enhancer; coordinates 5500939 to 5505948) expressed *lacZ* in leg imaginal discs. (D-G) Further sub-cloning of VLE produced overlapping 1 kb fragments VLE1 to VLE5 (in green) whose enhancer activity is shown in panels D-G. *lacZ* expression (red) was detected with anti-β-gal in third-instar leg imaginal discs co-stained with antibodies to the H15 (green) whose expression is the same as Mid. The single channel images (D′-G′) show the extent of the VLE expression domains. Discs are oriented dorsal up and anterior to the right throughout. Genotypes: D, *yw; VLE*; E, *yw; VLE1*; F, *yw; VLE4+5*; G, *yw; VLE5*.

The dorsal or ventral specific expression domains of genes controlling D/V pattern in the fly leg are maintained through a complicated genetic network involving indirect auto-regulation and negative feedback (Fig. 1A,B). Hh-signaling induces the dorsal signal *dpp* and the ventral signal *wg* (Basler and Struhl, 1994). *dpp* maintains its dorsal expression, in part, through the repression of ventral genes *wg*, *mid* and *H15* in dorsal cells; *wg* similarly maintains its Hh-dependent ventral expression domain by repressing dorsal genes including *dpp* and the downstream T-box gene *optomotor blind* (*omb*) (Brook and Cohen, 1996; Held and Heup, 1996; Jiang and Struhl, 1996; Johnston and Schubiger, 1996; Morimura et al., 1996; Penton and Hoffmann, 1996; Theisen et al., 1996). There are further layers of negative feedback. The downstream ventral T-box genes *mid* and *H15* are sufficient to repress dorsal genes and the dorsal T-box genes *omb* and *Dorsocross 1*, *2* and *3* (*Doc1*, *Doc2* and *Doc3*) are able to repress ventral genes (Reim et al., 2003; Svendsen et al., 2009). In this complex pathway it is difficult to discern how ventral specific *mid/H15* expression is regulated because many kinds of direct and indirect pathways are possible. For example, Dpp expression could repress *mid* from direct repressive action of Dpp pathway transcription factors, or indirectly through the activation of *omb*; alternatively, a combination of direct and indirect mechanisms could be acting.

In order to clarify how *mid* and *H15* expression is restricted to the ventral leg, we identified regulatory elements in the *mid* locus that respond to Wg and Dpp signaling as well as the downstream T-box genes, *omb*, *Doc3*, and *mid* itself. We also performed a simple lineage analysis of cells in the *mid* and *omb* expression domains and find that cells from the two domains do not mix during imaginal disc development.

## RESULTS

### Identification of a *midline* leg imaginal disc enhancer

For our studies of *mid* and *H15* gene regulation in the leg, we chose to focus our analysis solely on the regulation of *mid* because the two genes are expressed in identical patterns during leg disc development and because animals deleted for H15 are viable and have normal limb development (Svendsen et al., 2009). Previous work identified a GAL4 enhancer-trap, *mid*GAL4, inserted 23.6 kb downstream of *mid* (Hayashi et al., 2002) and expressed in a pattern very similar to both *mid* and *H15* (Svendsen et al., 2009). We reasoned the enhancer-trap, which detects the expression in the leg and antenna but not in other sites of *mid* expression, should lie close to a leg specific enhancer. We subdivided the 49 kb intergenic region 3′ of midline and 5′ of the adjacent downstream gene CG14020 into 5-7 kb fragments (Fig. 1C) using either convenient restriction sites or generating fragments through PCR amplification of a genomic BAC clone. We examined these fragments for reporter-expression during imaginal disc development and embryogenesis. All reporter constructs displayed complex patterns of expression expected for *mid* during embryogenesis including expression in tissues of the CNS, heart and ectoderm (not shown). Only one construct displayed expression in the ventral domain of the leg imaginal disc and so we named this element VLE for ventral leg enhancer. VLE was also expressed in a ventral wedge in the antennal disc but not in the wing, haltere or eye forming imaginal discs.

We compared the pattern of VLE expression to the distribution of H15 protein, which is expressed in an identical pattern to Mid in the leg, and for which a superior antibody is available (Svendsen et al., 2009). The domain of VLE was similar to H15 but with several differences (Fig. 1D). The lateral boundary of the VLE expression domain extends more broadly than H15 in the circumference of the disc along most of the proximal distal axis of expression. In the distal most region of the disc, VLE expression is less uniform and is expressed more weakly at the distal-most limit of H15 expression (Fig. 1D′). In the ventral proximal region the transgene expression is faithful to the antibody distribution, but does not show the expression in the disc stalk seen for H15 (and Mid) in the first leg pairs.

The functional significance of VLE is supported by a recent survey of chromatin structure in leg imaginal discs using the FAIRE technique (formaldehyde assisted identification of regulatory elements) (McKay and Lieb, 2013). FAIRE identifies chromatin in an open conformation and this study identified 57 FAIRE peaks in the entire genome that were specific to leg discs when compared to the peaks found in wing discs. The peaks were associated with 45 genes many of which are known to be key regulators of fly leg development. Two of these peaks are the only leg specific peaks in the *mid* region and overlap precisely with the ends of the VLE (Fig. S1).

### VLE and its derivatives are activated by Wg

**Fig. 2.**
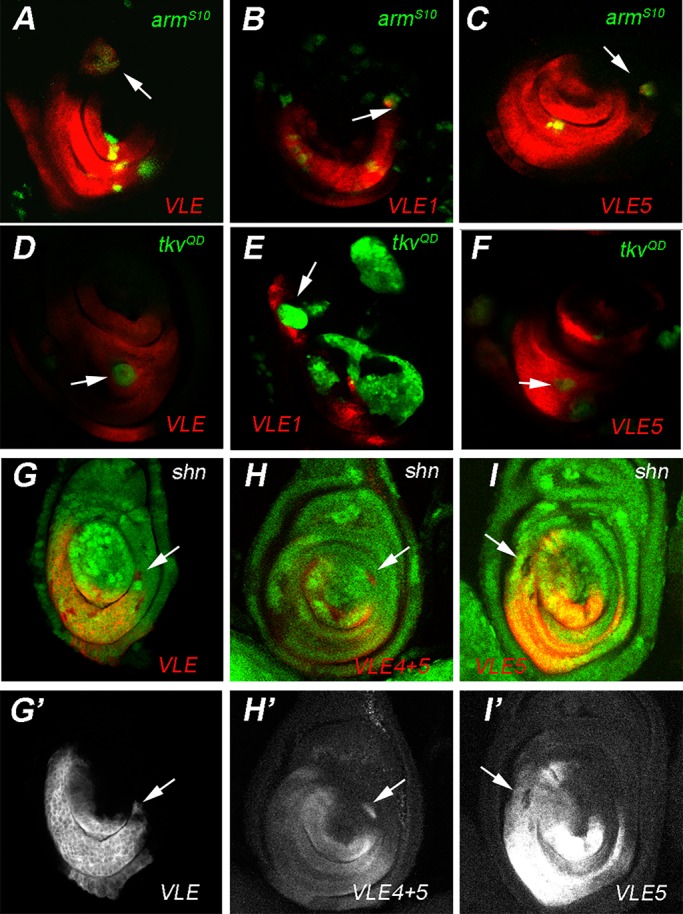
***mid* ventral leg enhancers are activated by Wg and repressed by Dpp.** Analysis of VLE-*lacZ* reporter gene expression (anti-β-gal, red) in clones expressing *arm^S10^* (green, A-C) or *tkv^QD^* (green, D-F), and in *shn^1B^* loss-of function clones (G-I). Activation of VLE (A), VLE1 (B), and VLE5 (C) in dorsal *arm^S10^* clones (seen as yellow) induced at 72-96 h ael (arrows). Repression of VLE (D), VLE1 (E) and VLE5 (F) in *tkv^QD^* expressing ventral clones induced at 48-72 h ael (arrows). VLE-*lacZ* expression in leg imaginal discs with *shn^1B^* loss-of-function clones (72-96 h ael) identified by loss of GFP expression. Ectopic expression of VLE (G,G′ arrow) and VLE4+5 (H,H′, arrow) is seen in *shn^1B^* LOF clones found outside of the expression domain. *shn^1B^* LOF clones in the VLE5 expression domain (I,I′, arrow) have decreased expression of VLE5 and clones outside this domain do not respond to loss of *shn*. Genotypes: A, *UAS-arm^S10^/yw; Ay-Gal4 UAS-GFP/+; VLE/hs-FLP[86E]*; B, *UAS-arm^S10^/yw; Ay-Gal4 UAS-GFP/+; VLE1*; C, *UAS-arm^S10^/yw; Ay-Gal4 UAS-GFP/+; VLE5*; D, *hs-FLP yw/yw; AY-Gal4 UAS-GFP/+;UAS-tkv^QD^; VLE/hs-FLP[86E]*; E, *hs-FLP yw/; AY-Gal4 UAS-GFP/+;UAS-tkv^QD^; VLE1*; F, *hs-FLP yw/; AY-Gal4 UAS-GFP/+;UAS-tkv^QD^; VLE 5/hs-FLP[86E]*; G, *hs-FLP yw; FRT42B shn^1B^/FRT42B Ubi-GFP; VLE/+*; H, *hs-FLP yw; FRT42B shn^1B^/FRT42B Ubi-GFP; VLE4+5/+*; I, *hs-FLP yw; FRT42B shn^1B^/FRT42B Ubi-GFP; VLE5/+*.

Since *mid* expression is regulated by Wg, we tested the effects of activating the Wg pathway on VLE expression. Cells expressing *arm^S10^* are constitutively activated for the Wg pathway (Pai et al., 1997). We expressed *arm^S10^* under GAL4/UAS control by generating clones of GAL4 expressing cells using the AyGAL4 flip-out cassette (Ito et al., 1997). We found that VLE was activated in a cell-autonomous manner in *arm^S10^* expressing clones. This was true of clones induced both in second instar larvae (48-72 h, not shown) and in third instar larvae (Fig. 2A). Clones located in the dorsal-most region of the leg imaginal disc did not tend to express VLE and this was consistent with H15 and Mid expression in *arm^S10^* clones (Svendsen et al., 2009). We attribute this effect to competing repression by dorsal factors such as Dpp. It is also possible that *mid* expression requires other positive inputs in addition to Wg, but these have not been identified.

In order to identify the regions required to drive ventral expression, we subdivided VLE into overlapping 1 kb fragments (Fig. 1C). VLE1 (Fig. 1E) and VLE5 (Fig. 1G) had ventral leg imaginal disc expression while VLE2, VLE3 and VLE4 had none (not shown). VLE1 expression is strong and extends further circumferentially than H15 in the proximal region. In the medial and distal domains VLE1 expression is more restricted than H15 and is weaker and more variable when compared to VLE. VLE5 had a much broader expression extending into the dorsal-lateral regions of the leg disc. Like VLE, VLE1 and VLE5 are each activated in *arm^S10^* clones (Fig. 2B,C).

### VLE and its derivatives are repressed by Dpp

The expression *mid* and *H15* is restricted from dorsal cells by Dpp signaling (Estella and Mann, 2008; Svendsen et al., 2009). This regulation does not involve the Dpp target gene *brinker* (Estella and Mann, 2008). In order to test whether Dpp signaling represses VLE, we expressed a construct encoding a constitutively activated *thick veins (tkv^QD^)* receptor (Lecuit, et al., 1996) also using AyGAL4. Virtually all *tkv^QD^*-expressing clones induced at 48-72 h in the ventral expressing regions of VLE and VLE1 had reduced lacZ expression. VLE5 expression is also lost in *tkv^QD^*-expressing clones only less frequently (Fig. 2D,E,F; Table S1). We confirmed the gain-of function results by looking at clones mutant for the Dpp pathway element *Mothers-against-decapentaplegic* (*Mad*), an R-Smad required for both activation and repression functions of Dpp (Affolter et al., 2001). We induced clones of a strong hypomorphic allele, *Mad^1.2^* and chose the 72-96 h window because, prior to 72 h, Dpp signaling is required in proximal distal patterning (Galindo et al., 2002). We found cell-autonomous ectopic expression of VLE, VLE1 and VLE5 in some *Mad^1.2^* clones situated outside the reporter gene expression domains (Fig. S2A-C). In order to ensure that the ectopic expression of the reporter genes was due to loss of Dpp signaling and not due to Wg activation in *Mad* clones, we also tested expression in clones doubly mutant for *Mad* and *wg* and found that VLE, VLE1 and VLE5 were still expressed ectopically (Fig. S2E-G). Furthermore, we did not detect ectopic Wg expression in a sample of *Mad* clones labeled with anti-Wg (not shown). In the ventral domain, we observed that VLE, VLE1 and VLE5 reporter expression was largely unaffected in *Mad^1.2^* mutant clones except for subtle increases in VLE expression (Table S1). The lack of effect on VLE expression in the ventral domain also shows that *Mad* is not required for Wg target gene expression, unlike the expression of some Wg target genes in the wing that do depend on Mad (Zeng et al., 2008; Eivers et al., 2009).

### The VLE4 fragment contains a Schnurri-responsive element

Taken together our data show that like Mid and H15 expression, VLE is restricted from dorsal expression by Dpp signaling. Canonical repression by Dpp signaling requires the transcriptional co-repressor encoded by *schnurri* (*shn*)*.* Following phosphorylation of Mad by activated Tkv, Shn binds with Mad and Medea (Med), a fly co-smad, and the complex represses Dpp target genes (Pyrowolakis et al., 2004). We reasoned that if VLE was a direct target of Dpp repression, then removing *shn* function in clones of cells should result in autonomous increases in reporter gene expression. Consistent with a requirement for direct repression by Dpp, *shn* mutant cells located outside the expression domain frequently have cell autonomous ectopic VLE expression (9/45) (Fig. 2G,G′). All clones ectopically expressing VLE were located in the distal or medial regions of the imaginal discs. In contrast, *shn* clones outside the reporter gene expression domain did not cause any ectopic expression of VLE1 (0/17 clones) or VLE5 (0/35 clones) (Fig. 2I,I′), indicating that neither construct responds to direct repression by Dpp signaling. Similarly, some clones caused weak increases in VLE-expressing cells but this was not observed in VLE1 or VLE5. The lack of de-repression of VLE1 and VLE5 suggests that elements required for *shn*-mediated repression of VLE lie in between VLE1 and VLE5. We tested this by combining the VLE4 and VLE5 fragments in a construct, VLE4+5 (Fig. 1C,F), and found that VLE4+5 had expression and response to manipulation of Dpp signaling that was similar to VLE. It was repressed by *tkv^QD^* expression (Fig. S2H) and activated in *Mad* (Fig. S1D) and *shn* clones (Fig. 2H,H′), confirming that the VLE4 fragment is required for *shn* mediated Dpp repression.

We also noted that a majority of *shn* clones located in the VLE5 expression domain had either a cell-autonomous reduction or loss of expression, which is the opposite of what would be predicted for a repressor of *mid* expression (Fig. 2I). A smaller fraction of VLE and VLE4+5 clones have a similar effect. The decreased expression may be due to occasional reduced viability in *shn* clones. Alternatively, the result may indicate that *shn* may have a complex role, acting in both repression and, unexpectedly, in activation of the *mid* enhancer through the VLE5 fragment.

### The *mid* leg enhancer is repressed by the Dpp target genes *Doc3* and *omb*

Our results suggest that the VLE1, 4, and 5 regions all respond to Dpp signaling but only the VLE4 region responds to the *shn*-dependent repressor complex of the Dpp pathway. Since Dpp signaling can either activate or repress target genes, the repression of gene expression by Dpp signaling could be both through the activation of downstream repressors and through *shn*-dependent repression. The dorsal T-box genes *omb* and *Doc1*, *2* and *3* are excellent candidates to mediate indirect repression of *mid* by Dpp. The *omb* expressing domain is similar to that of the region of elevated dorsal *dpp* expression, a thin strip of cells that run along the dorsal anterior/posterior (A/P) boundary, abutting but not overlapping the *mid* domain (Svendsen et al., 2009); *Doc3* expression is restricted to a region of the dorsal leg proximal to the tibia (Reim et al., 2003). Both genes have CREs that contain canonical Dpp-activation motifs and so are likely activated directly by Dpp (Weiss et al., 2010). To test whether the *mid* CREs were responsive to *omb* or *Doc*, we induced AyGAL4 *Doc3*- and *omb-*expressing clones at 72-96 h after egg laying (ael). We found that the expression of *lacZ* is either reduced or lost altogether in the *Doc3* expressing clones induced in the ventral domains of H15, VLE, VLE1, VLE4+5, and VLE5 (Fig. S3A,A′-E,E′; Table S1). All constructs also responded to ectopic *omb* expression at 72 to 96 h, with many ventral clones losing reporter expression (Fig. S3F,F′-J,J′; Table S1).

### Mid and H15 expression is subject to negative feedback

**Fig. 3.**
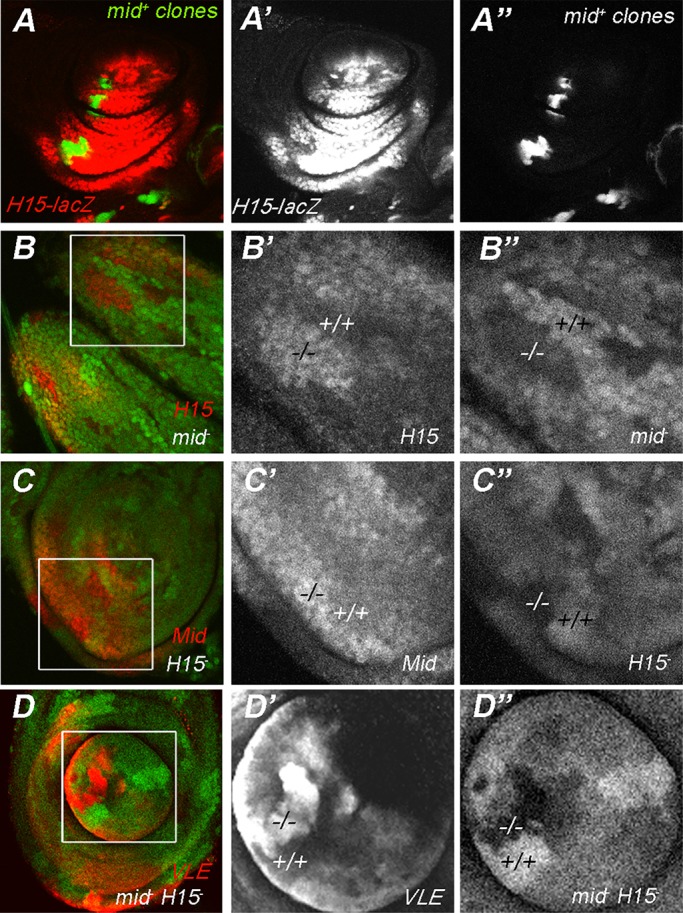
**Mid and H15 cross-regulation.** Overexpression of *mid* in ventral GAL4 expressing clones (seen as GFP+ patches, A″) causes autonomous decrease of *H15-lacZ* expression (A′). Clones homozygous for the *mid^1^* null allele (GFP negative cells in a heterozygous GFP background, B″ −/−) have increased H15 expression (red, or in the single channel, B′ −/−). Cells in the twin-spot are homozygous for *mid*^+^ (indicated by two copies of GFP, B″ +/+) and have decreased H15 expression (B′ +/+). The reverse experiment is seen in C. Clones homozygous for the *H15^X4^* null allele are GFP− (C,C″ −/−) have increased *mid* expression (C red, C′ −/−); again, the twin-spots (H15^+/+^) show decreased *mid* expression (C′ +/+). *H15^X4^mid^1a5^* double mutant clones (lack of GFP; D,D″−/−) express higher autonomous levels of VLE (D red, D′). Twin-spots, which have wild type levels of H15 and mid (+/+), show decreased levels of *lacZ* expression (D′ +/+). Genotypes: A, *yw hs-FLP/ yw; AY-Gal4 UAS-GFP/H15-lacZ b^1^ cn^1^; UAS-mid2.12*; B, *yw hs-FLP/yw; mid^1^ FRT40A/Ubi-GFP FRT40A*; C, *yw hs-FLP/ yw; H15^X4^ FRT40A/ Ubi-GFP FRT40A*; D, *yw hsFLP/yw; H15^X4^ mid^1a5^ FRT40A/Ubi-GFP FRT40A; VLE*.

Our previous study suggested that *mid* may regulate *H15* expression because ectopic expression of *mid* in the ventral leg autonomously reduced the expression of an H15 reporter gene (Svendsen et al., 2009) (Fig. 3A). We tested whether loss of function had a reciprocal effect and found that H15 protein levels were increased in *mid^1^* null clones (Fig. 3B) compared to the levels of expression in the surrounding *mid^+^* heterozygous tissue. Furthermore, H15 levels were decreased in the adjacent homozygous *mid^+^* twin spots (Fig. 3B,B′). We found similar results for Mid levels in *H15* mutant cells, with Mid increased in somatic clones homozygous for the *H15^X4^* null allele and decreased in wild type twins (Fig. 3C,C′) although this result is not as clear because the quality the Mid antisera is not as good as that for H15. These two results suggest that each gene is sensitive to a 2-fold change in dosage of the other. We extended this analysis by looking at the VLE constructs in double mutant *H15^X4^ mid^1a5^* clones. Like both H15 and Mid, VLE (Fig. 3D,D′) showed increased expression in *H15 mid* loss-of-function clones and was down-regulated in *H15^+^mid^+^* twins (Fig. 3D,D′). We saw similar results for VLE1 and similar but less consistent effects for VLE4+5, while VLE5 did not respond to loss of *H15* and *mid* function (Table S1). Together this data suggests that in addition to activation by Wg and repression by Dpp, proper *mid* and *H15* expression levels are maintained through negative autoregulation.

### Restriction of cell mixing between ventral and dorsal cells

**Fig. 4.**
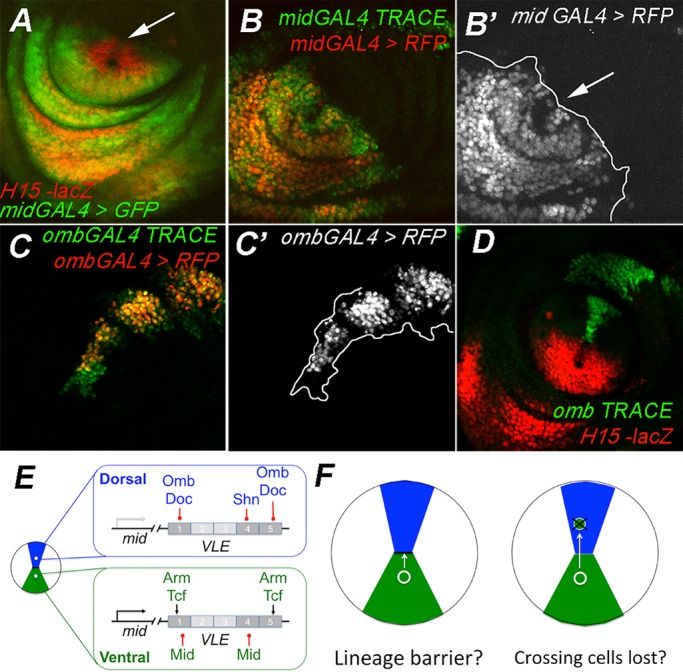
***mid*Gal4 and *omb*Gal4 expressing cells do not mix during disc development.** (A) The distal-most domains of the *midGal4* and *H15-lacZ* expression domains are aligned (arrow). (B) *mid*GAL4 expression, indicated by RFP (red) and clones of GFP^+^ (green) cells induced in the *mid*GAL4 domain throughout disc development. The line in B′ denotes the extent of the *mid*Gal4 lineage compared to the *mid*GAL4 expression indicating that clones on the lateral edges migrate outside the *mid*GAL4 expression domain but the distal-most clones do not (arrow). (C) The same experiment using the *omb*Gal4 driver shows that GFP^+^ clones (green) born in the *omb*GAL4 domain do migrate outside of the *omb*GAL4 expression domain marked with RFP (red). The extent of the clone migration is outlined in C′. (D) Clones born in the *omb*GAL4 domain do not extend into the H15 domain (*H15-lacZ*, red), whose boundary matches *mid* lineage distally. (E) The model depicts the potential interactions of transcription factors with *mid* leg enhancer regions of the *mid* locus in dorsal (blue) and ventral (green) leg imaginal disc cells such that the mid gene is OFF dorsally and ON ventrally. (F) Possible models to explain lack of mixing of dorsal and ventral cells. Genotypes: A, w*; NP2113 midGal4*/H15-lacZ b^1^ cn^1^*; *UAS-GFP(S65T)*; B, w*; UAS-RedStinger[4] UASFLP1.D[JD1] Ubi-p63E(FRT.STOP)Stinger[9F6]/NP2113 midGal4; C, *omb*Gal4/w*; UAS-RedStinger[6] UAS-FLP.Exel[3] Ubi-p63E(FRT-STOP)Stinger[15F2]/+; D, *omb*Gal4/w*; H15-lacZ b^1^ cn^1^/+; UAS-Flp.exel[3] Ubi-p63E9(FRT.STOP)Stinger[15F2]/+.

Our data indicate that restriction of the expression of *mid* to the ventral domain depends on Dpp signaling pathway transcription factors and transcription factors downstream of Dpp. The ventral expression domain of *mid* and the dorsal domain of *omb* expression are non-overlapping during larval development from as early as late-second instar stage larvae (Fig. S4A). If transcriptional repression downstream of Dpp is the sole mechanism that restricts *mid* and *wg* expression to ventral cells, then cells may be expected to traverse freely between the dorsal and ventral domains, changing gene expression depending on their position. In order to test whether cells do migrate between the D/V expression domains we used the G-trace system (Evans et al., 2009). This technique labels all cells that have expressed GAL4 by inducing stable GFP expressing clones while simultaneously labeling cells that currently express GAL4 with UAS-RFP. To examine ventral cells, we used *mid*GAL4 expression to induce clones. The lateral margins of *mid*GAL4 expression extend further out than the endogenous Mid domain, but critically for this experiment, the distal most edge of *mid*GAL4 expression coincides with the distal edge of H15 and Mid (Fig. 4A). Inducing GFP-expressing clones with G-trace under the control of *mid*GAL4, we find that clones induced in the distal most part of the *mid*Gal4 domain, i.e. the cells adjacent to the *omb/dpp* cells, do not extend dorsally beyond the current Gal4 expressing domain as indicated with RFP (Fig. 4B,B′; *n*=12). This was not the case in more lateral cells, where GFP expressing cells do extend several cell diameters beyond *mid*GAL4 expressing cells. This suggests that *mid*-expressing cells near the interface with *dpp* and *omb* expressing cells are somehow excluded from more dorsal positions while more lateral *mid*GAL4 cells are not. We did the reciprocal experiment testing whether cells expressing *omb*GAL4 exited the dorsal domain during leg development. In most cases, we observed GFP-expressing clones located outside the *omb*GAL4 expression domain, indicating that cells expressing *omb*GAL4′ do leave the expression domain (Fig. 4C,C′). In order to determine whether these cells entered the *mid* expression domain, we induced GFP expressing clones in the *omb* domain and compared their location with an H15 reporter that is expressed in the same cells as Mid and H15. In this experiment, we found no overlap of GFP and *lacZ* (red, Fig. 4D) in a sample of 13 imaginal discs. Thus, the cells that exit the *omb* domain are still excluded from the *mid* domain. The G-trace results suggest that in addition to repression by dorsal genes (Fig. 4E), the expression domain of *mid* may be maintained by cellular mechanisms that prevent the mixing of ventral *mid* expressing cells and dorsal *omb* expressing cells (Fig. 4F).

## DISCUSSION

The correct patterning of the dorsal ventral axis of the *Drosophila* leg depends on the maintenance of discrete dorsal and ventral domains of morphogen and selector gene expression. By using loss- and gain-of-function genetic assays, our lab and others have identified multiple possible pathways of cross repression between dorsal and ventral genes that maintain dorsal/ventral (D/V) gene expression patterns (Brook and Cohen, 1996; Jiang and Struhl, 1996; Johnston and Schubiger, 1996; Lecuit et al., 1996; Morimura et al., 1996; Penton and Hoffmann, 1996; Theisen et al., 1996; Svendsen et al., 2009) (Fig. 1B). In this report, we identified and characterized VLE, a ventral leg enhancer in the *mid* selector gene locus. In order to refine our understanding of how *mid* is regulated, we tested the effects of genetic manipulation of Wg, Dpp and downstream dorsal and ventral genes on the expression of VLE and VLE sub-fragments. Using this approach, we were able to identify the regions within VLE that are responsible for activation by Wg and repression by Dpp. Our data suggest that *mid* expression in the ventral domain is maintained through a combination of Wg activation and negative autoregulation by *mid* and *H15*. Further, our data suggest that in dorsal cells, Dpp signaling represses VLE by two pathways. The first pathway requires the co-repressor *shn* while the second pathway does not require *shn* and may act indirectly with Dpp activating the repressors *omb* and *Doc* (Fig. 4E). Finally we show that ventral and dorsal cells from the *mid* and the *omb/dpp* expression domains do not mix during larval development indicating that ventral specific *mid* expression may also be maintained by cellular mechanisms (Fig. 4F).

Several lines of evidence suggest that VLE is an important enhancer element in the *mid* region. The VLE expression pattern is similar to *mid* and responds to the same regulatory manipulations as the endogenous *mid* gene. VLE is the only region in ∼45 kb of 3′ flanking-DNA tested in this study that could drive reporter expression in leg imaginal discs. Furthermore, previous work (McKay and Lieb, 2013) demonstrated a remarkable correspondence between a leg disc-specific open chromatin conformation detected by FAIRE and the sub-regions of VLE implicated in regulation of *mid* expression in this study. While these results indicate that VLE is a key regulatory element for *mid* leg expression, differences between VLE and *mid* in expression pattern and timing suggest that additional regulatory elements are likely required to fully reproduce wild-type *mid* expression. VLE has a good correspondence to mid in the distal domain, but in more proximal regions it has a broader expression. This could indicate that VLE is more sensitive to activation by Wg, less sensitive to the effects of Dpp repression, or perhaps cells might maintain VLE expression longer than endogenous *mid* expression. Another difference between VLE and *mid* or *H15* expression is the timing of the response to Dpp signaling. VLE and its derivatives are de-repressed in response to loss of Dpp in early third instar (72-96 h) more frequently than endogenous *H15* (and presumably *mid*). There is direct evidence for at least one other limb enhancer in the *mid* 3′ regulatory region. Another study that surveyed regulatory elements across the entire genome identified a second enhancer in the *mid* 3′ regulatory region that drives expression in a restricted portion of the ventral leg (Jory et al., 2012). This region is located near the *mid*GAL4 insertion site, in a region from which we were not able to recover transformants (PCR0, Fig. 1C). Thus, the region flanking the *mid*GAL4 insertion, the VLE region described here, and perhaps other unknown elements, may all be required to recapitulate precise *mid* expression.

Despite the differences in pattern and timing between VLE and endogenous *mid* expression, our results have clarified several aspects of the D/V pathway with respect to *mid* regulation. Expression in the ventral domain is mediated by Wg activation, as expected, but our work also shows that ventral expression is modulated by negative autoregulation by *mid* and *H15*. The feedback of *mid* on its own expression suggests that limiting mid levels may be important for ventral leg development. A role for negative autoregulation by the repressor Snail (Sna) been proposed as a mechanism for promoting the uniform *sna* expression required in presumptive mesoderm during gastrulation (Boettiger and Levine, 2013). Negative autoregulation by Mid may act to promote a more uniform expression of *mid* in response to a graded input of Wg signaling, with higher levels of Wg incapable of promoting higher Mid because of negative feedback.

Also, as expected, VLE and its sub-fragments respond to changes in Dpp signaling and to the downstream T-box repressors *Doc3* and *omb*. These results suggest a pathway of repression where Dpp acts to repress *mid* indirectly through the activation of *omb* and *Doc3.* A second direct pathway for Dpp repression of *mid* is supported by the ectopic expression of VLE4 containing constructs in cells lacking *shn*. Thus our results suggest a model where the activation of *mid* by Wg is antagonized by multiple pathways of repression including negative autoregulation, direct repression by Dpp signaling and feed-forward repression by Dpp through activation of downstream dorsal genes. It is quite possible that all of the genes in the D/V pathway will have similarly complex gene regulation and that this complexity may indeed contribute to the stability of D/V expression domains.

The effects on *mid* expression described by our genetic analysis do not necessarily indicate direct binding of any of these factors to sequences in VLE, but it is the most straightforward interpretation. Sequence analysis of VLE (not shown) indicates several possible binding sites for T-box factors and Tcf sites required for Wg activation based on published consensus sequences (Chang et al., 2008; Najand et al., 2012; Jin et al., 2013). Repression by the Shn/Mad/Med complex has been shown to work through a GRCGNC(NNNNN)GTCTG motif (Pyrowolakis et al., 2004) and two near matches for this sequence are located in VLE4 (data not shown). We also searched the entire *mid* 3′ region and found one exact match for the canonical Dpp repression site located outside the VLE fragment, 2 kb closer to the *mid* transcription unit. However, the significance of this sequence is not clear because a 10 kb fragment including both the predicted site and the entire VLE fragment did not give substantially different expression pattern compared to VLE in reporter assays (data not shown). Further analysis will be required to determine whether any of the predicted sites in VLE play a functional role in regulating *mid* expression.

The ventral specific expression of *mid* may also be maintained by the lack of mixing between dorsal and ventral cells at the distal end of the *mid* expression domain. We found that clones of cells born in the ventral *mid* expression domain were not recovered in the dorsal *omb* domain and vice versa. This suggests that the gene expression patterns maintained through complex transcriptional cross-repression may be further reinforced by cellular mechanisms. Several possible mechanisms include lineage barriers, loss of cells that migrate into the wrong domain, and oriented cell division patterns that prevent cells from moving into the adjacent domain (Fig. 4F). We favor a lineage barrier mechanism because studies have shown that clones of cells activated for the Wg or Dpp pathways tend to form smooth boundaries with neighboring cells when the clones are located in the opposite territory (Diaz-Benjumea and Cohen, 1994; Jiang and Struhl, 1996; Heslip et al., 1997). The smooth boundaries suggest that cells in the clone minimize contact with surrounding cells and this behavior can be indicative of a lineage barrier (Dahmann et al., 2011). Lineage barriers are also known to maintain the expression boundaries of other limb selector genes. The boundary between posterior *engrailed* (*en*) expressing cells and anterior cells in all fly limbs is controlled by differential Hh signaling and *en* activity (Blair and Ralston, 1997; Rodriguez and Basler, 1997; Dahmann and Basler, 2000). These differences result in differential mechanical tension at the A/P boundary that restricts cells from crossing (Landsberg et al., 2009). A similar lineage boundary exists in the wing imaginal disc at the interface between dorsal *apterous* expressing cells and ventral cells and is mediated by Notch signaling (Micchelli and Blair, 1999; Rauskolb et al., 1999). The A/P boundary of the leg and wing and the wing D/V boundary each span hundreds of cell diameters and were detected first with classical lineage experiments using genetic mosaics for adult cuticle markers (Garcia-Bellido et al., 1973; Dahmann et al., 2011). Similar experiments did not detect any distinct boundaries between the dorsal and ventral domains of the leg (Steiner, 1976). However, this is not surprising due to the technical limitations of the approach and the small interface between the dorsal and ventral gene expression domains of the leg that spans only a few cells. Further work will be required to determine how dorsal and ventral cells are prevented from mixing and what genetic elements of the D/V pathway are required to maintain the segregation of dorsal and ventral cells.

## MATERIALS AND METHODS

### *Drosophila* stocks

Flies were grown under standard conditions at 25°C. *midline* 3′enhancer(VLE)-*lacZ* stocks are described below; other stocks were obtained from Bloomington Indiana, Kyoto Stock Centers, Sean Carroll (*FRT42D shn^1B^*), or have been previously described (Lecuit et al., 1996; Buescher et al., 2004; Svendsen et al., 2009).

### Reporter constructs from the *mid* 3′ regulatory region

Standard techniques were used to subclone regions 3′ to the *midline* gene. To obtain some fragments, PCR was performed using BAC R44J04 (*Drosophila melanogaster* chromosomal coordinates 2L:5389189-5554072) as template. The fragments were subcloned upstream of *lacZ* in the pH-pelican vector and standard P-element-mediated germ-line transformation into *y^1^ w^1118^* flies was performed using Delta2-3 as helper plasmid (Robertson et al., 1988). Transgenics were tested for *lacZ* expression to identify fragments driving expression in larval leg discs. The VLE fragments generated and used in this study are VLE (2L: 5500939-5505948), VLE1 (2L: 5500939-5502008), VLE2 (2L: 5501959-5503038), VLE3 (2L: 5502979-5504058), VLE4 (2L: 5503999-5505078), VLE4+5 (2L: 5503999-5505948) and VLE5 (2L: 5505019-5505948). The sequence coordinates of other cloned fragments can be provided upon request.

### Genetic mosaics, ectopic expression and lineage analysis

*Mad^1.2^*, *Mad^1.2^ wg^cx4^*, and *shn^1b^* loss-of-function clones were generated as previously described (Xu and Rubin, 1993; Svendsen et al., 2009) at 48-72 or 72-96 h ael by crossing female P{ry^+t7.2^=hsFLP}1, y^1^ w*; P{ry^+t7.2^=neoFRT}42D P{w^+mC^=Ubi-GFP(S65T)nls}2R/CyO flies to males of genotype *y^1^ w^1118^;P{ry^+t7.2^=neoFRT}42D shn^1B^/CyO* and carrying an insert of an *mid* VLE-*lacZ* construct or, females of genotype P{ry^+t7.2^=hsFLP}1, y^1^ w*;P{w^+mC^=Ubi-GFP(S65T)nls}2L P{ry^+t7.2^=neoFRT}40A to males of genotype *y^1^ w^1118^; Mad^1.2^ P{ry^+t7.2^=neoFRT}40A/CyO* or *y^1^ w^1118^; Mad^1.2^ wg^cx4^ P{ry^+t7.2^=neoFRT}40A/CyO* and also carrying an insert of a VLE-*lacZ* construct to monitor expression in clones lacking GFP. Clones expressing *UAS-arm^S10^* (constitutively active in Wg signaling) (Pai et al., 1997), *UAS-tkv^QD^* (constitutively active in Dpp signaling) (Lecuit et al., 1996), *UAS-Doc3* (Reim et al., 2003) and *UAS-omb4.15* (Grimm and Pflugfelder, 1996) were induced using the y^1^ w*; P{w^+mC^=AyGAL4}25 P{w^+mC^=UAS-GFP.S65T}Myo31DF[T2] driver in a line also carrying a VLE-*lacZ* construct (Ito et al., 1997). For lineage analysis, G-Trace stocks (Evans et al., 2009) w*; P{w^+mC^=UAS-RedStinger}4, *P{w^+mC^=UAS-FLP1.D}JD1,* *P{w^+mC^=Ubi-p63E(FRT.STOP)Stinger}9F6* or w*; P{w^+mC^=UAS-RedStinger}6, *P{w^+mC^=UAS-FLP.Exel}3,* *P{w^+mC^=Ubi-p63E(FRT.STOP)Stinger}15F2* (from Bloomington) were crossed to *mid*GAL4=w*; *P{w^+mW.hs^=GawB}NP2113/CyO* or *omb*GAL4=*P{GawB}bi^md653^, y^1^ w^1118^/FM7*. For studies comparing *mid* expression to *omb* lineage, the strains used were *P{GawB}bi^md653^, y^1^ w^1118^*, TRACE G0 (w*; P{w^+mC^=UAS-Flp.exel}3, *P{w^+mC^=Ubi-p63E (FRT.STOP) Stinger}15F2*) and w*; *H15-lacZ*=*PZ[lacZ;ry+]H15 b^1^ cn^1^*.

### Immunohistochemistry

Discs were stained using the protocol of Pattatucci and Kaufman (1991). Primary antibodies used were rabbit anti-Nmr1 (H15; 1/500) and anti-NMR2 (mid, 1:500 provided by Jim Skeath, Washington University School of Medicine, St. Louis, MO, USA; Leal et al., 2009), mouse anti-β-Gal (1:1000; Promega), rabbit anti-β-gal (1;1000; Jackson ImmunoResearch Laboratories). Fluorescent detection was performed with Alexa Fluor-labeled secondary antibodies (1:500; Molecular Probes, Inc.).
